# Supplementary material for: Urban pollution greatly enhances formation of natural aerosols over the Amazon rainforest
Source: Nat Commun. 2019 Mar 5;10:1046. doi: 10.1038/s41467-019-08909-4 (PMC6401186; doi:10.1038/s41467-019-08909-4)
Supplement: Supplementary file 1 — Supplementary Information [file 41467_2019_8909_MOESM1_ESM.pdf]

Supplementary Information for

Urban pollution greatly enhances formation of natural aerosols over the Amazon rainforest

Shrivastava *et al.*

[*] *To whom correspondence should be addressed. Email: ManishKumar.Shrivastava@pnnl.gov*

<u>**Supplementary Figures**</u>

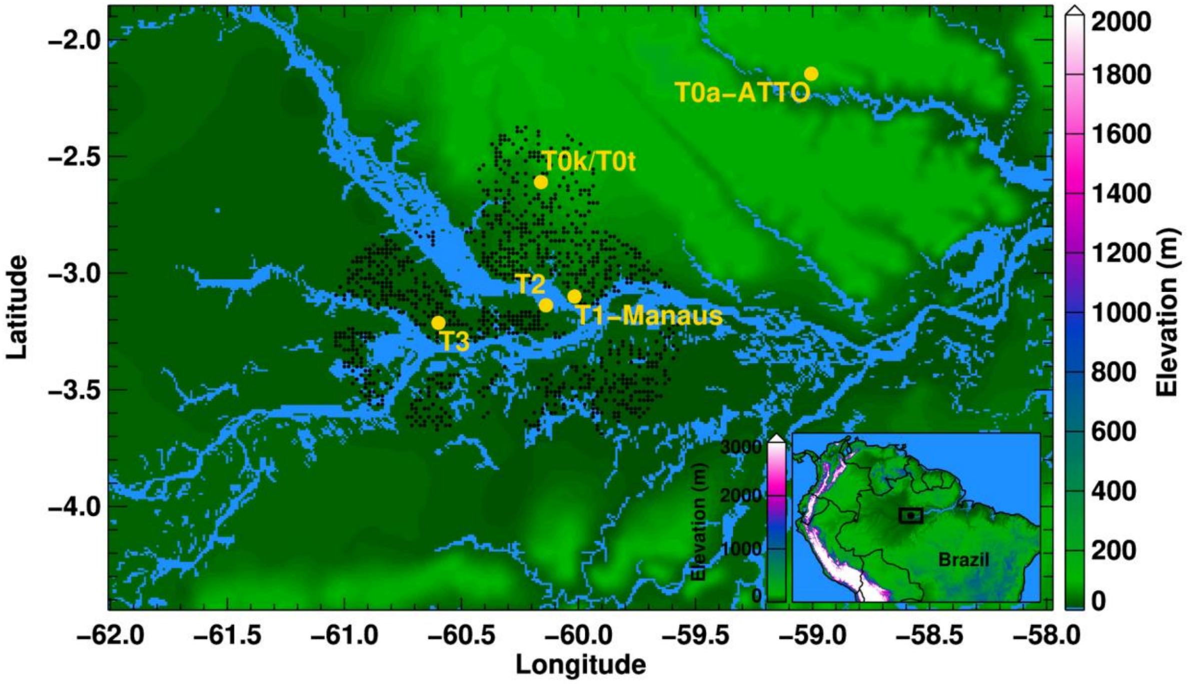

Supplementary Figure 1: WRF-Chem simulated inner nested domain (2 km grid spacing). The black rectangle in the inset figure shows the location of this domain in South America. Surface measurement sites are shown in yellow dots, while black dots represent random sampling points in the model, chosen for comparison with observed meteorological parameters at measurement sites as described in Methods.

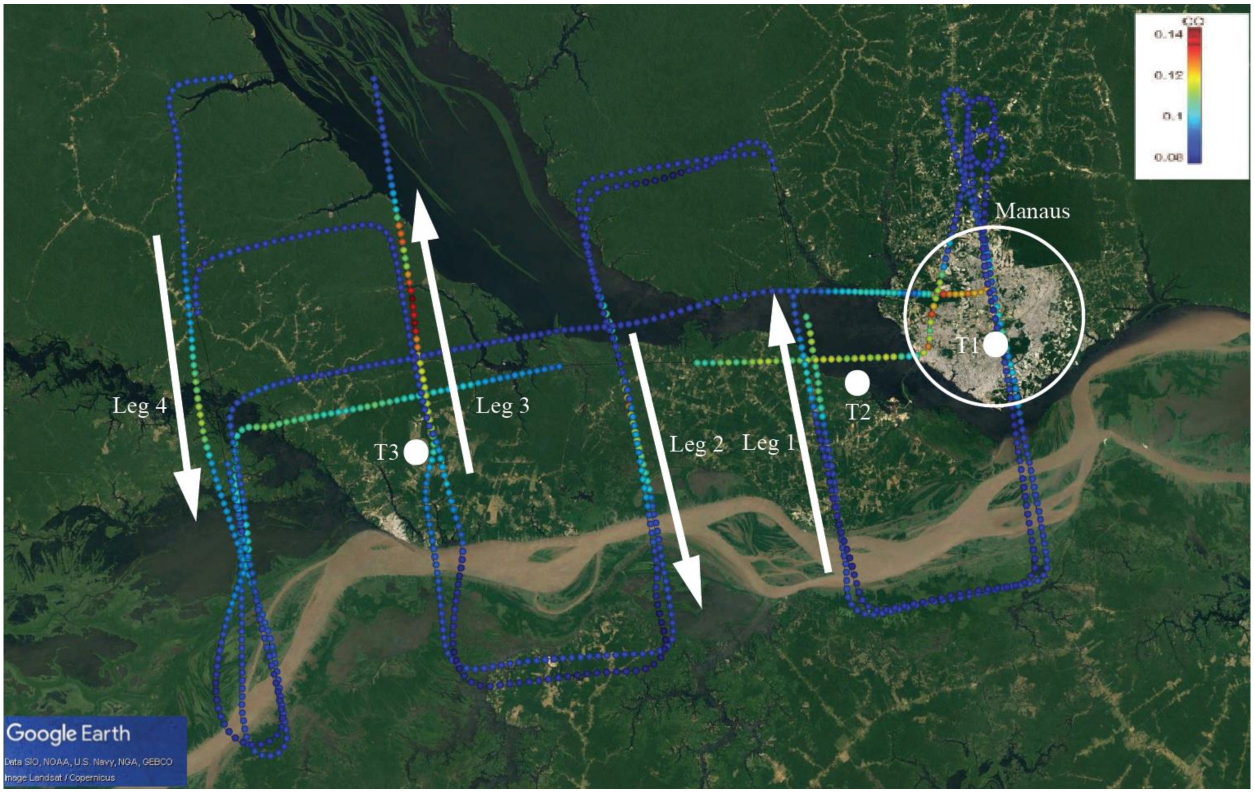

Supplementary Figure 2: Aircraft flight legs on March 13, 2014 colored by measured CO concentrations that indicate location of the Manaus plume. Arrows indicate directions of the flight. Legs 1-4 are aircraft transects (spaced ~24 km apart), plotted in Figure 1a in the main text. The white filled circles show locations of the ground measurement sites: T1 is within the Manaus urban area, T2 and T3 are located downwind of Manaus.

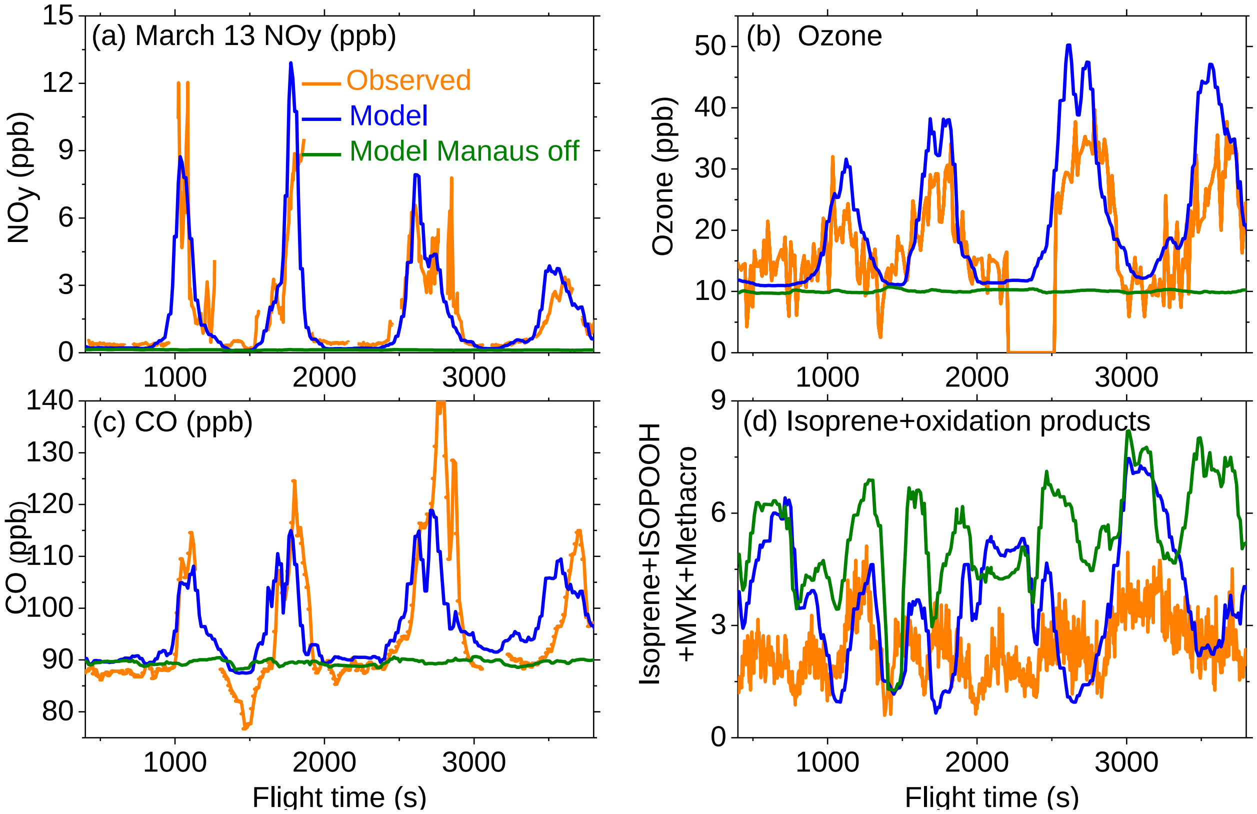

Supplementary Figure 3: Measured (orange) and model predicted values for simulations with all emissions on (blue), and biogenic emissions on but Manaus emissions turned off (green). Values are plotted along aircraft flight transects at 500-m altitude on March 13 (a) NOy (b) Ozone (c) CO and (d) sum of isoprene and its first generation oxidation products: ISOPOOH, methyl vinyl ketone (MVK) and methacrolein.

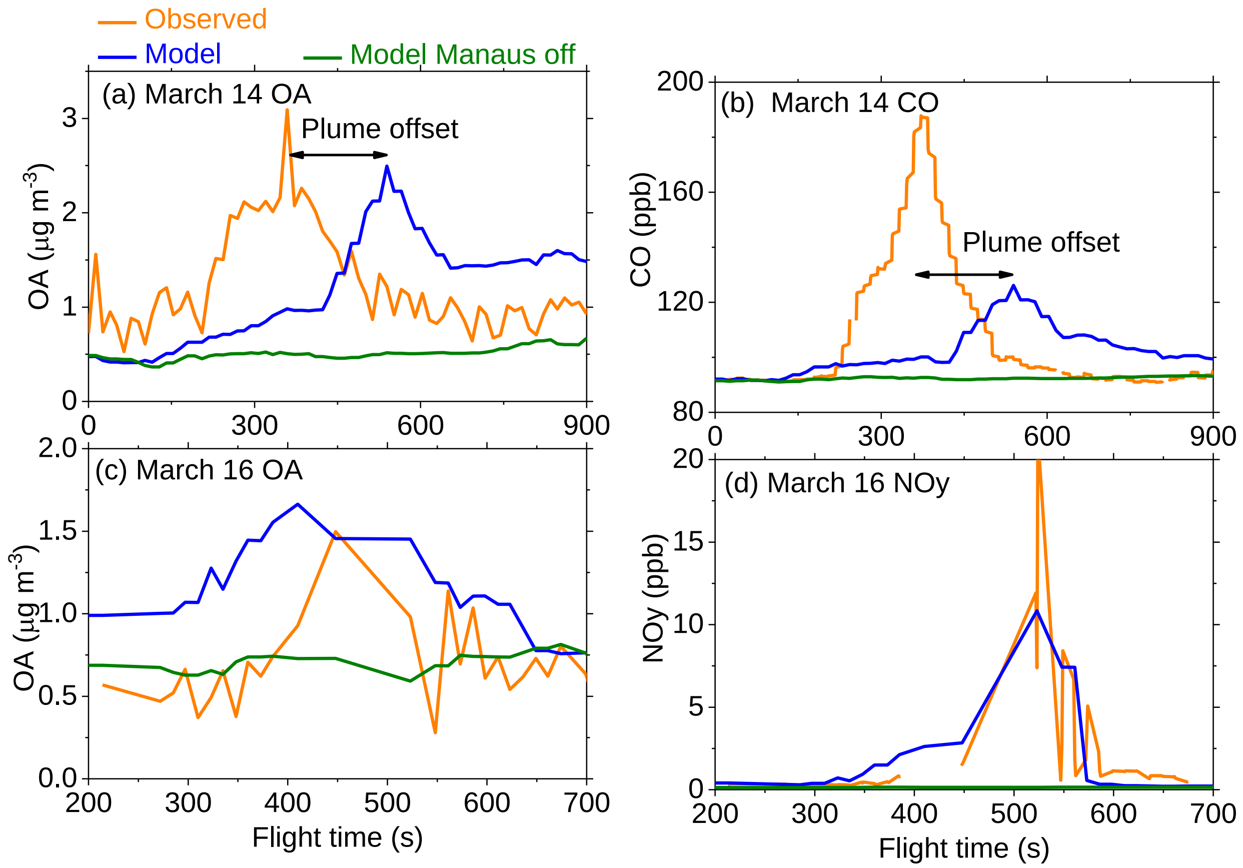

Supplementary Figure 4: Measured (orange) and model predicted values for simulations with all emissions on (blue), and biogenic emissions on but Manaus emissions turned off (green). Values are plotted along aircraft flight transects at 500-m altitude**.** (a) and (b): March 14, (c) and (d): March 16. (a) & (d) show OA on March 14 and 16, respectively. (b) CO and (d) NOy indicate how the locations of measured and simulated plumes compare on these days.

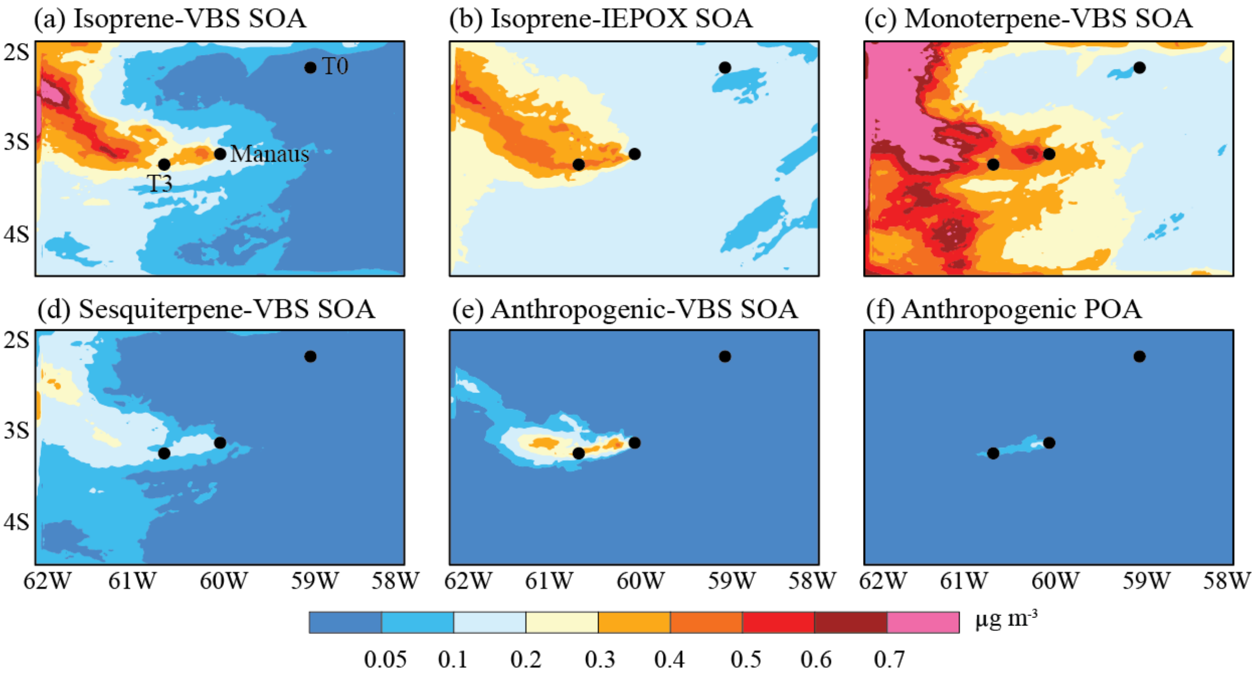

Supplementary Figure 5: **Simulated OA component concentrations (μg m^{-3}) at 500 m altitudes, averaged during the afternoons (16-20 UTC) of March 13, 2014** (a) Isoprene-VBS SOA (pure gas-phase chemistry pathway) (b) Isoprene IEPOX-SOA (multiphase chemistry pathway using simpleGamma model) (c) Monoterpene-VBS SOA (d) Sesquiterpene-VBS SOA (e) Anthropogenic-VBS SOA (f) Anthropogenic POA, as described in Methods.

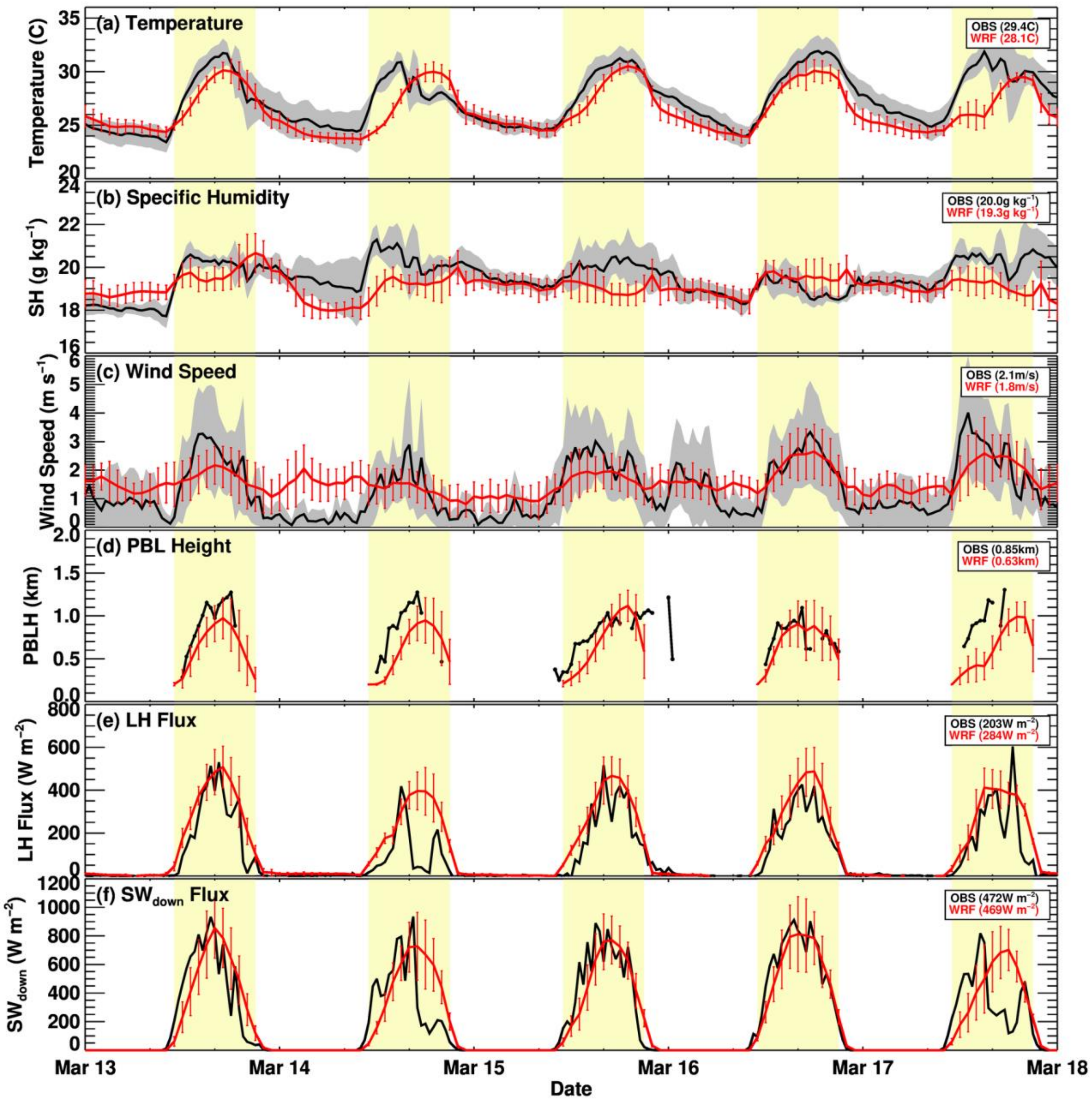

Supplementary Figure 6: **Observed and WRF-Chem simulated variations of meteorological fields over the simulated domain** (a) 2 m air temperature (b) 2 m specific humidity (c) 10 m wind speed (d) planetary boundary layer (PBL) height (e) latent heat (LH) flux (f) surface downwelling shortwave (SW_{down}) radiation. The model (red) reasonably simulates variations in these meteorological fields over the domain compared to observations (black). Observed temperature, specific humidity, wind speeds (a-c) are from T1-Manaus, T2, and T3 sites, gray shadings are standard deviations from the 3 sites, error bars are standard deviations from the WRF-Chem simulated mean values. PBL heights (d) and SW_{down} radiations (f) are from the T3 site, latent heat fluxes are from T0k site. Although WRF-Chem simulations are done for March 10-17, results are only plotted from March 13-17, since the first 3 days are used for model spin-up.

Supplementary Tables

Supplementary Table 1: WRF-Chem model configuration

WRF domain	10 km resolution outer domain covering and 2 km resolution nested domain.
Simulation period	March 10-17, 2014
Boundary layer	YSU scheme
Surface	Community Land Model (CLM v4)
Cloud microphysics	Morrison (2 moments) [1, 2]
Radiation	RRTMG scheme (both longwave and shortwave)
Gas-phase photochemistry	SAPRC-99
Aerosol chemistry	MOSAIC for inorganic aerosols<br>Modified Volatility basis set (VBS) for organic aerosols as described in the text
Land use	Community land model (CLM) with US Geological Survey (USGS) dataset. CLM is run at the same resolution as WRF-Chem.
Boundary conditions	NCEP CFS reanalysis for meteorology and quasi-global WRF for aerosols and trace gases [3]

Supplementary Table 2: Biogenic SOA yields derived from fitting chamber data using a volatility basis set VBS (C^* = 0.1, 1, 10 and 100 µg m^{-3}), which defines the first few generations of oxidation products of isoprene, monoterpenes, and sesquiterpenes.

Precursor	Oxidant	Low NO_x yields for C^* (µg m^{-3})				High NO_x yields for C^* (µg m^{-3})			
		0.1	1	10	100	0.1	1	10	100
Isoprene	OH	0.0[a]	0.01[a]	0.036[a]	0.008[a]	0.0[b]	0.019[b]	0.063[b]	
	ozone			0.012[c]					
Monoterpene	OH		0.275[d]	0.10[d]	0.17[d]		0.034[d]	0.199[d]	
	ozone	0.08[e]	0.019[e]	0.18[e]	0.03[e]	0.08[e]	0.019[e]	0.18[e]	0.03[e]
Sesquiterpene	OH		0.013[d]	0.805[d]				1.215[d]	
	ozone	0.014[a]	0.052[a]	0.046[a]	0.0[a]	0.014[a]	0.052[a]	0.046[a]	0.0[a]
		Nitrate radical (NO_3) yields							
Isoprene	NO_3		0.001[f]	0.149[f]	0.237[f]				
Monoterpene	NO_3	0.373[g]	0.033[g]		0.941[g]				
Sesquiterpene	NO_3	0.373[g]	0.033[g]		0.941[g]				

Data from which above yields are derived: [a]ref [4], [b]ref [5], [c]ref [6], [d]ref [7], [e]ref [8], [f]ref [9], [g]ref[10].
Molecular weights of all V-SOA species are assumed to be 250 g $mole^{-1}$.

Supplementary Table 3: Aircraft-measured and WRF-Chem simulated median values of absolute OA concentrations in-plume and at background locations. Calculations are shown for individual flight transects on 13 March 2014, since the aircraft clearly identified the plume evolution on this day, as described in the text. An average across all flight transects is shown for March 14 and 16, and the modeled plume was determined by scanning all radial locations downwind T1 site, as described in the main text.

OA	Measured OA (µg m^{-3})		Model OA (µg m-3)		Percent Enhancement (Plume-Background)/Background*100	
Flight Transects March 13	In-plume	Background	In-plume	Background	Measured	Model
1	1.2	0.5	1.0	0.5	140	111
2	1.4	0.5	1.5	0.5	180	192
3	1.6	0.5	1.6	0.5	220	228
4	1.3	0.5	1.9	0.5	160	283
Flight March 14	2.0	0.9	1.5	0.5	122	199
Flight March 16	1.0	0.6	1.4	0.7	67	101

Supplementary Table 4: Aircraft-measured and WRF-Chem simulated median values of absolute ozone concentrations in-plume and at background locations on 13 March 2014.

OA	Measured Ozone (ppb)		Model Ozone (ppb)	
Flight Transects March 13	In-plume	Background	In-plume	Background
1	19.8	13.8	25.4	9.9
2	24.4	13.7	32.5	10.0
3	32.1	11.2	38.8	10.1
4	29.0	10.5	36.1	9.9
March 14	29.1	15.6	40.1	8.9
March 16	18.2	15.0	17.2	10.4

Supplementary Table 5: Reactions added to SAPRC-99 gas-phase chemistry mechanism to include gas-phase chemistry of IEPOX and isoprene dihydroxy dihydroperoxide $ISOP(OOH)_2$

No.	Reactions forming IEPOX	A (cm^3 $molecule^{-1}$ s^{-1})	Ea/R (K)
1	ISOPRENE + OH = ISOPOO +additional species	2.5E-11	-408
2	ISOPOO + HO_2 = HO_2[a]+ 0.88 ISOPOOH	7.40E-13	-700
3	ISOPOO + NO = NO[a] + volatile products[b]	2.7E-12	-350
4	ISOPOO + ISOPOO = volatile products[b]	7.00E-16	
5	ISOPOOH + OH = OH[a] + 0.75IEPOX+0.13ISOPOO+0.12$ISOP_2O_2$	1.90E-11	-390
6	IEPOX + OH = OH[a] + volatile products[b]	5.78E-11	400
	Competing reactions forming $ISOP(OOH)_2$		
1	$ISOP_2O_2$ + HO_2 = $ISOP(OOH)_2$ + HO_2[a]	7.4E-13	-700
2	$ISOP_2O_2$+NO = volatile products +NO[a]	2.7E-12	-350
3	$ISOP_2O_2$ = isomerization products[b] with isomerization rate = 0.3 s^{-1} based on [11]		

[a]species are recycled because we don't want to change the radical budgets in the parameterized SAPRC-99 mechanism, [b]no explicit products are considered. Reaction rate constants for isoprene oxidation products with HO_2 and OH are from Paulot et al.[12], while those for reactions with NO are generic constants already included in SAPRC-99 for oxidation of isoprene. Gas-phase IEPOX is then input to the simpleGamma model formulation to predict IEPOX-SOA formation.

Supplementary References

1. Morrison H, Curry JA, Khvorostyanov VI. A new double-moment microphysics parameterization for application in cloud and climate models. Part I: Description. *Journal of the Atmospheric Sciences* **62**, 1665-1677 (2005).

2. Morrison H, Thompson G, Tatarskii V. Impact of Cloud Microphysics on the Development of Trailing Stratiform Precipitation in a Simulated Squall Line: Comparison of One- and Two-Moment Schemes. *Monthly Weather Review* **137**, 991-1007 (2009).

3. Hu ZY, *et al.* Trans-Pacific transport and evolution of aerosols: evaluation of quasi-global WRF-Chem simulation with multiple observations. *Geosci Model Dev* **9**, 1725-1746 (2016).

4. Chen Q, Liu Y, Donahue NM, Shilling JE, Martin ST. Particle-Phase Chemistry of Secondary Organic Material: Modeled Compared to Measured O:C and H:C Elemental Ratios Provide Constraints. *Environ Sci Technol* **45**, 4763-4770 (2011).

5. Chan A, *et al.* Role of aldehyde chemistry and NOx concentrations in secondary organic aerosol formation. *Atmos Chem Phys* **10**, 7169-7188 (2010).

6. Kleindienst TE, Lewandowski M, Offenberg JH, Jaoui M, Edney EO. Ozone-isoprene reaction: Re-examination of the formation of secondary organic aerosol. *Geophysical Research Letters* **34**, (2007).

7. Ng NL, *et al.* Effect of NO(x) level on secondary organic aerosol (SOA) formation from the photooxidation of terpenes. *Atmos Chem Phys* **7**, 5159-5174 (2007).

8. Shilling JE, *et al.* Particle mass yield in secondary organic aerosol formed by the dark ozonolysis of alpha-pinene. *Atmos Chem Phys* **8**, 2073-2088 (2008).

9. Ng NL, *et al.* Secondary organic aerosol (SOA) formation from reaction of isoprene with nitrate radicals (NO(3)). *Atmos Chem Phys* **8**, 4117-4140 (2008).

10. Boyd CM, *et al.* Secondary organic aerosol formation from the beta-pinene+NO3 system: effect of humidity and peroxy radical fate. *Atmos Chem Phys* **15**, 7497-7522 (2015).

11. D'Ambro EL, *et al.* Isomerization of Second-Generation Isoprene Peroxy Radicals: Epoxide Formation and Implications for Secondary Organic Aerosol Yields. *Environ Sci Technol* **51**, 4978-4987 (2017).

12. Paulot F, *et al.* Unexpected Epoxide Formation in the Gas-Phase Photooxidation of Isoprene. *Science* **325**, 730-733 (2009).
